# Supplementary material for: Poly-ligand profiling differentiates trastuzumab-treated breast cancer patients according to their outcomes
Source: Nat Commun. 2018 Mar 23;9:1219. doi: 10.1038/s41467-018-03631-z (PMC5865185; doi:10.1038/s41467-018-03631-z)
Supplement: Supplementary file 1 — Supplementary Information(PDF 3592 kb) [file 41467_2018_3631_MOESM1_ESM.pdf]

Poly-ligand profiling differentiates trastuzumab-treated breast cancer patients according to their outcomes

Valeriy Domenyuk[1], Zoran Gatalica[1], Radhika Santhanam[1], Xixi Wei[1], Adam Stark[1], Patrick Kennedy[1], Brandon Toussaint[1], Symon Levenberg[1], Jie Wang[1], Nianqing Xiao[1], Richard Greil[2], Gabriel Rinnerthaler[2], Simon P. Gampenrieder[2], Amy B. Heimberger[3], Donald A. Berry[4], Anna Barker[5], John Quackenbush[6], John L. Marshall[7], George Poste[1,5], Jeffrey L. Vacirca[8], Gregory A. Vidal[9], Lee S. Schwartzberg[9], David D. Halbert[1], Andreas Voss[1], Daniel Magee[1], Mark R. Miglarese[1], Michael Famulok*[1,10,11,12], Günter Mayer*[1,10,12], David Spetzler*[1]

[1]*Caris Life Sciences, 4610 South 44th Place, Phoenix, AZ 85040, USA*

[2] *IIIrd Medical Department, Oncologic Center, Paracelsus Medical University Salzburg, Austria and Salzburg Cancer Research Institute, and Cancer Cluster Salzburg, Müllner Hauptstraße 48A-5020 Salzburg, Austria*

[3]*Dept. of Neurosurgery, University of Texas MD Anderson Cancer Center, 1400 Holcombe Blvd, Houston, TX 77030, USA*

[4] *Berry Consultants, LLC, 3345 Bee Caves Rd, Suite 201, Austin, Texas 78746, USA*

[5]*Complex Adaptive Systems Initiative, Arizona State University, 1475 N. Scottsdale Rd., Suite 361, Scottsdale, AZ 85257, USA*

[6]*Dept. of Biostatistics and Computational Biol., Dana-Farber Cancer Institute, 450 Brookline Ave. Boston, MA 02215-5450, USA*

[7]*Dept. of Oncology, Lombardi Comprehensive Cancer Center, Georgetown University Medical Center, 3800 Reservoir Road, N.W., Washington D.C. 20057, USA*

[8]*North Shore Hematology Oncology Associates Cancer Center, 226 N Belle Mead Rd., East Setauket, NY 11733, USA*

[9]*Division of Hematology and Oncology, University of Tennessee Health Science Center, 7945 Wolf River Blvd. Memphis, TN 38138, USA*

[10]*LIMES Program Unit Chemical Biology & Medicinal Chemistry, c/o Kekulé Institute for Organic Chemistry and Biochemistry, University of Bonn, Gerhard-Domagk-Straße 1, 53121 Bonn, Germany*

[11]*Chemical Biology Max-Planck-Fellowship Group, Center of Advanced European Studies and Research (CAESAR), Ludwig-Erhard-Allee 2, 53175 Bonn, Germany*

[12]*Center of Aptamer Research and Development, University of Bonn, University of Bonn, Gerhard-Domagk-Straße 1, 53121 Bonn, Germany*

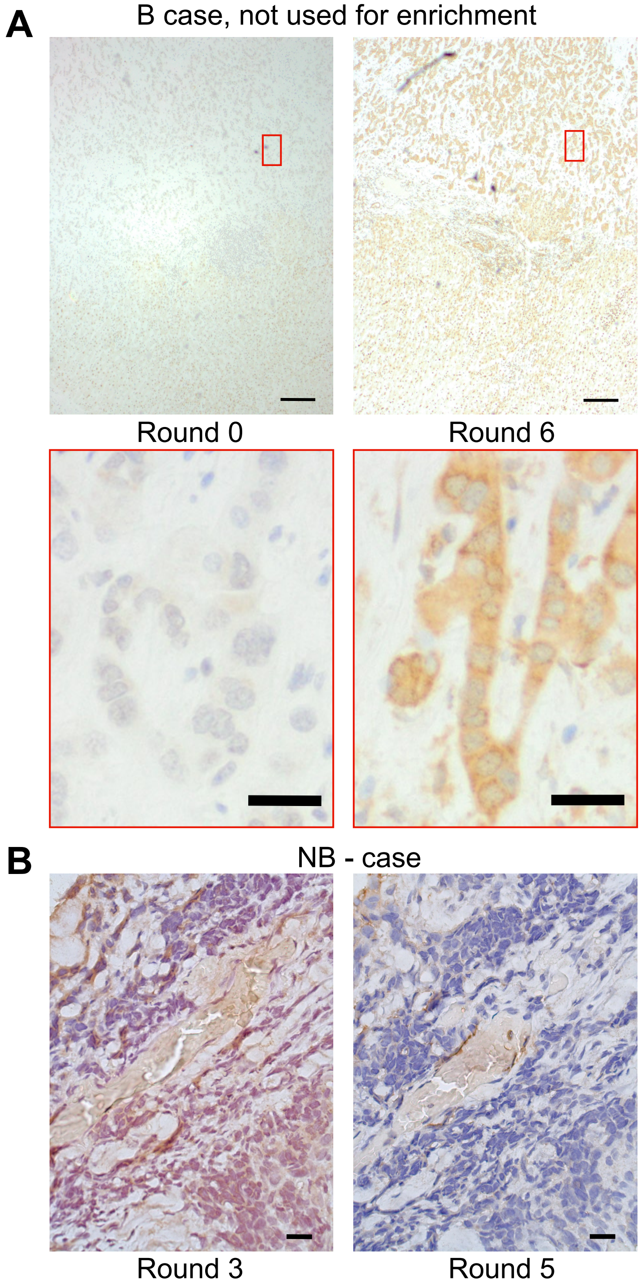

Supplementary Figure 1. Staining of library EL-B on benefiter and non-benefiter tissues. **A**. Staining of tissue from a B case not used during the selection process with unenriched library (round 0), compared to the enriched library EL-B (round 6; upper panel: 4×, 200 µm scale bar, lower panel: 60×, 10 µm scale bar). **B**. Staining of the tissue of a patient who did not derive benefit from C+T regimens (NB) by polyligand profiling (PLP) and employed for counter selection in the enrichment of EL-B, using the output ssDNAs from round 3 (left), compared to the output ssDNAs from round 5 (right) (20×, 40 µm scale bar). No adjustment was applied to images from microscopy.

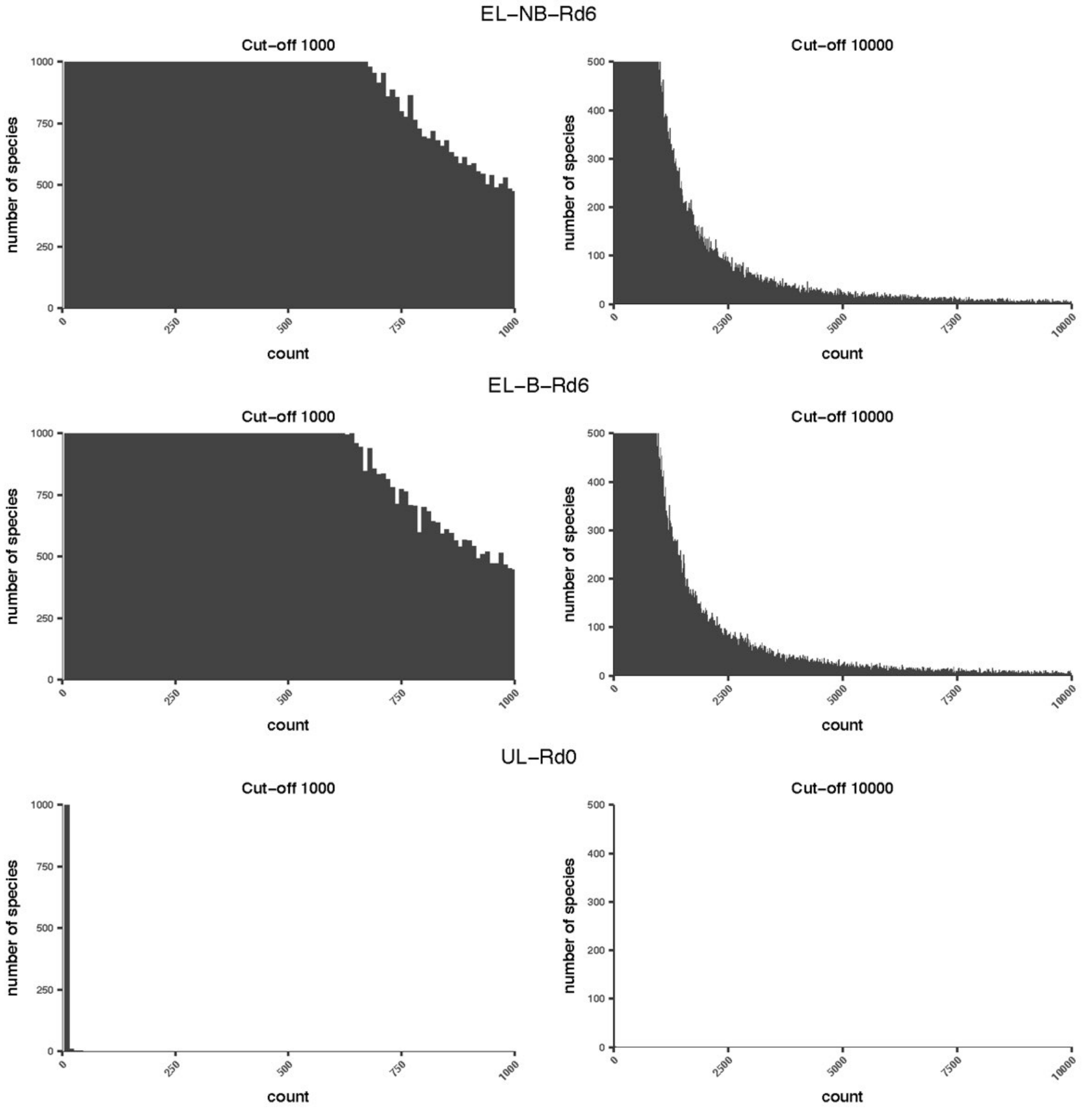

Supplementary Figure 2. Number of unique sequences (species) at different cut-offs based on the read counts obtained by NGS of the enriched post-round 6 libraries EL-NB-Rd6 (upper panel) and EL-B-Rd6 (middle panel) as well as unenriched starting library UL-Rd0 (lower panel). All panels on the left side have X axes restricted to 1000 counts to demonstrate that the UL-Rd0 library sequences have very low counts before the enrichment. All panels on the right have X axes expanded to 50000 to demonstrate the distribution of the counts for >99.9% of species in the enriched libraries. For better visualization, the y axes are restricted to 1000 species in the left panel and to 500 species in the right panels.

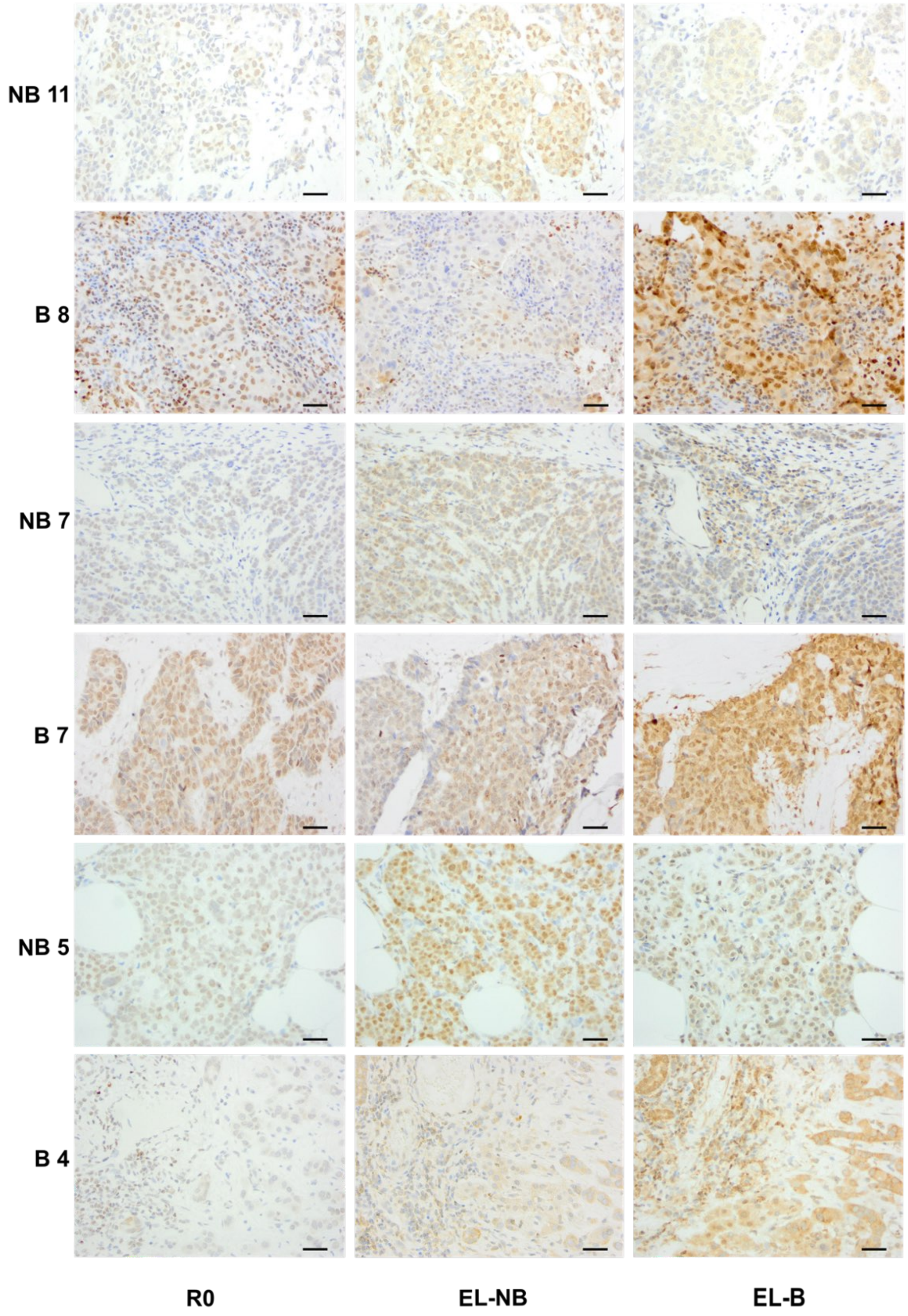

Supplemetary Figure 3, part 1.

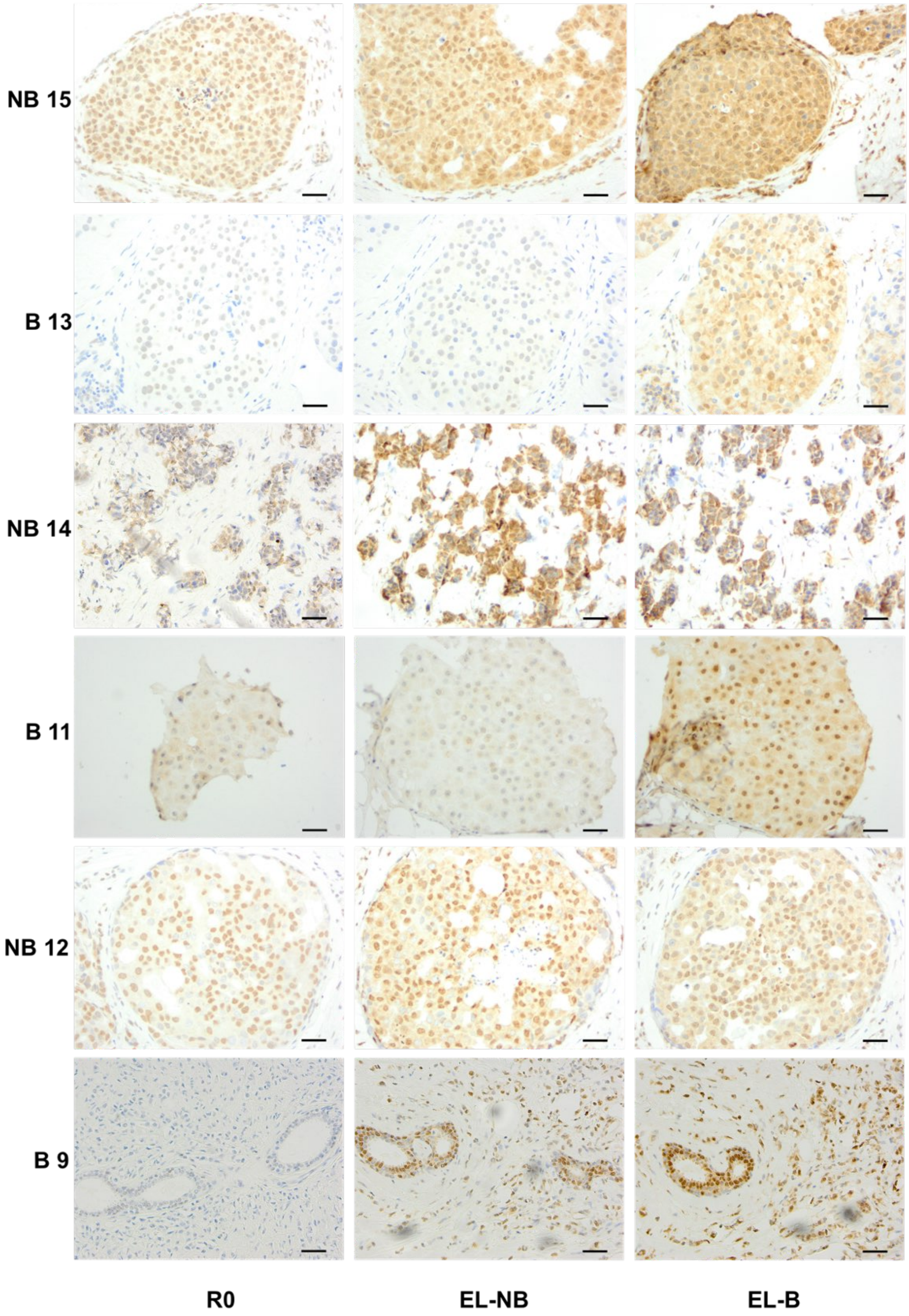

Supplementary Figure 3, part 2.

Supplementary Figure 3. Poly-Ligand Profiling (PLP) staining profiles comparison of the non-enriched starting library (R0) and enriched libraries EL-NB and EL-B on patients not benefiting (NB) and benefiting (B) from C+T or T treatment. View areas are matched in each row between libraries within each panel. The library R0 usually exhibits little to no staining, while the enriched libraries can be scored from 1+ to 3+. Library EL-NB, which was enriched toward a C+T non-benefiting case, exhibits stronger intensity on NB cases, except for NB-15. Library EL-B, which was enriched toward a C+T benefiting case, exhibits stronger intensity on the B cases. Magnification: 20×; 40 µm scale bar. No adjustment was applied to images from microscopy.

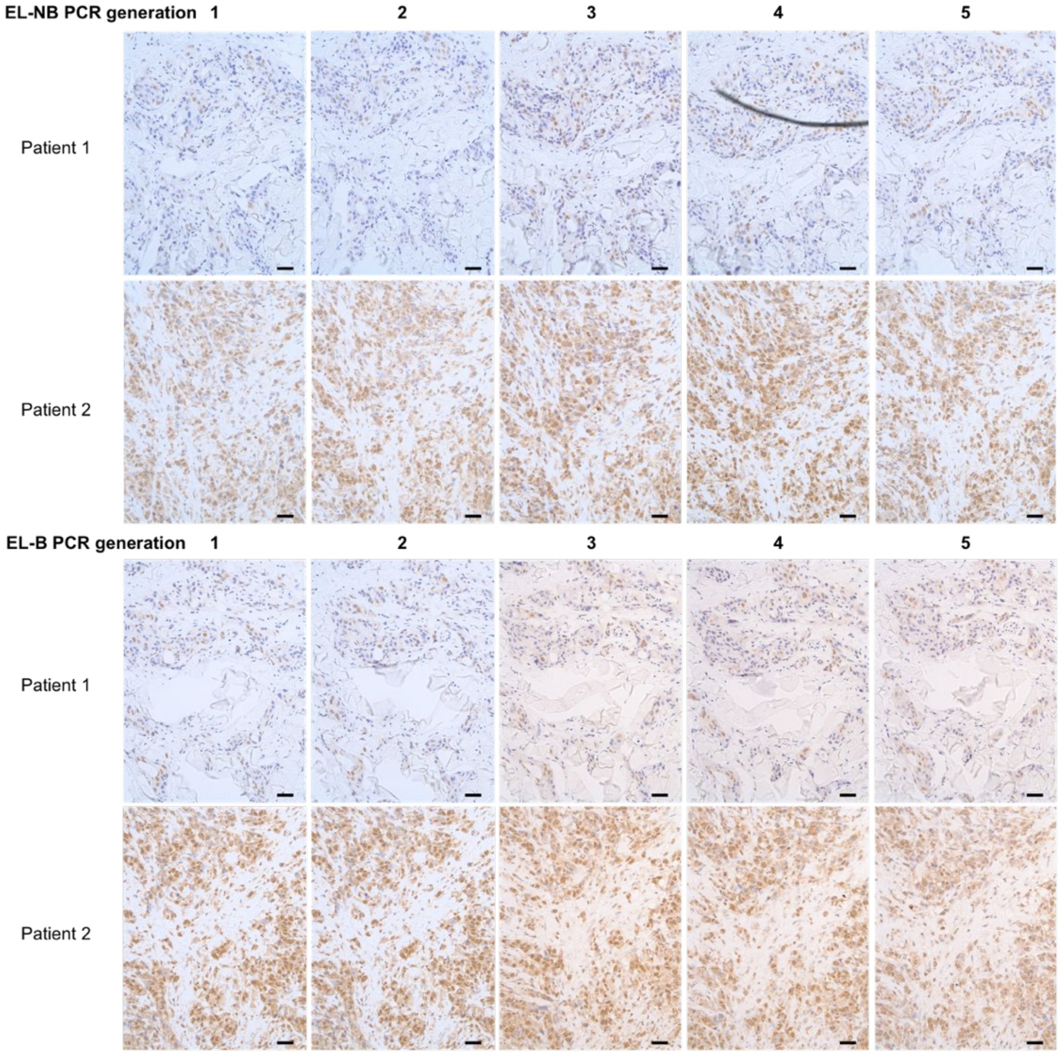

Supplementary Figure 4A, part 1.

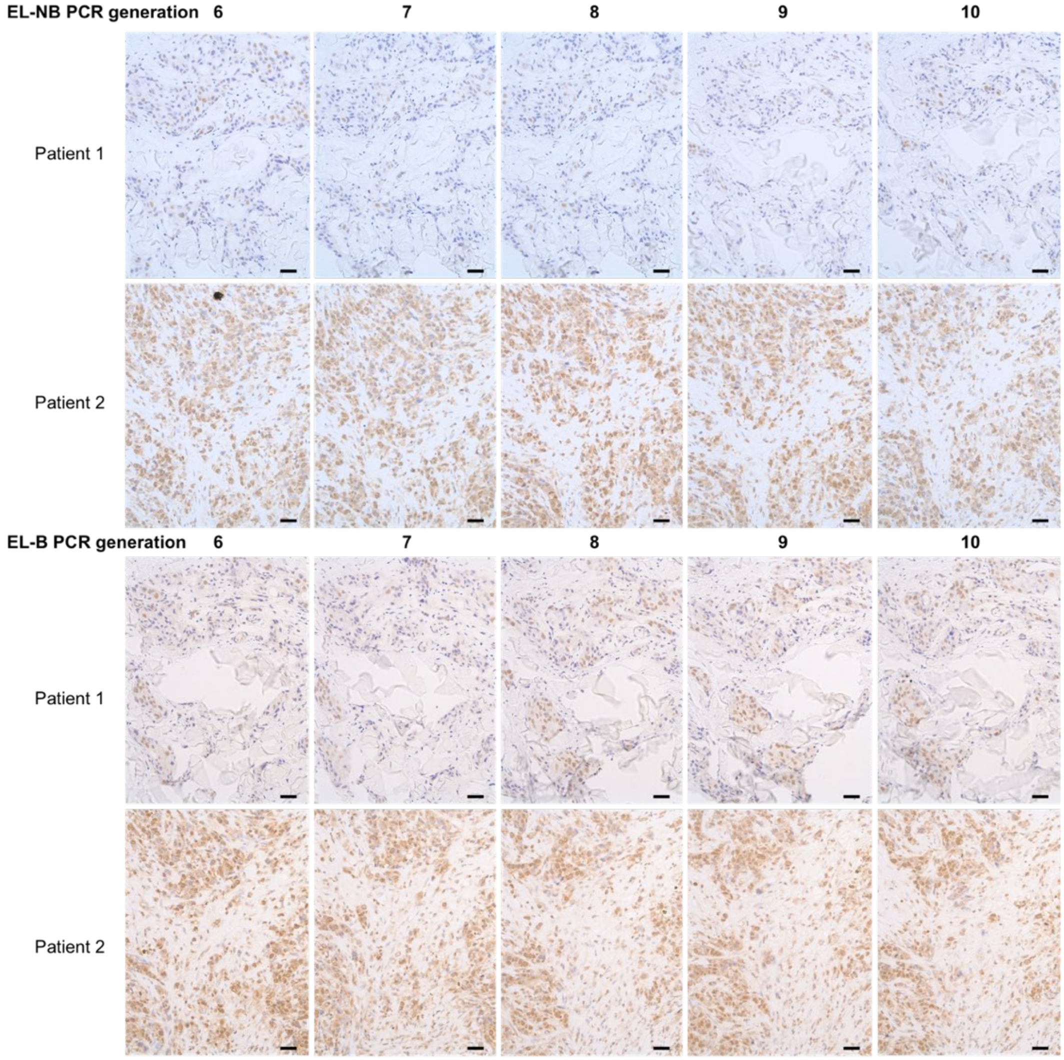

Supplementary Figure 4A, part 2.

Supplementary Figure 4A. Technical reproducibility of the staining with libraries EL-NB and EL-B resulting from different PCR-generations 1-5 (part 1), and 6-10 (part 2). Examples from two different cases at 20× magnifications are shown, 40 µm scale bar. For each PCR generation, an aliquot (0.4 ng) of each preceding library generation was amplified for 10 PCR cycles and ssDNA was purified. Each PCR generation of EL-NB and EL-B was then used for the staining of consecutive tissue sections. No adjustment was applied to images from microscopy.

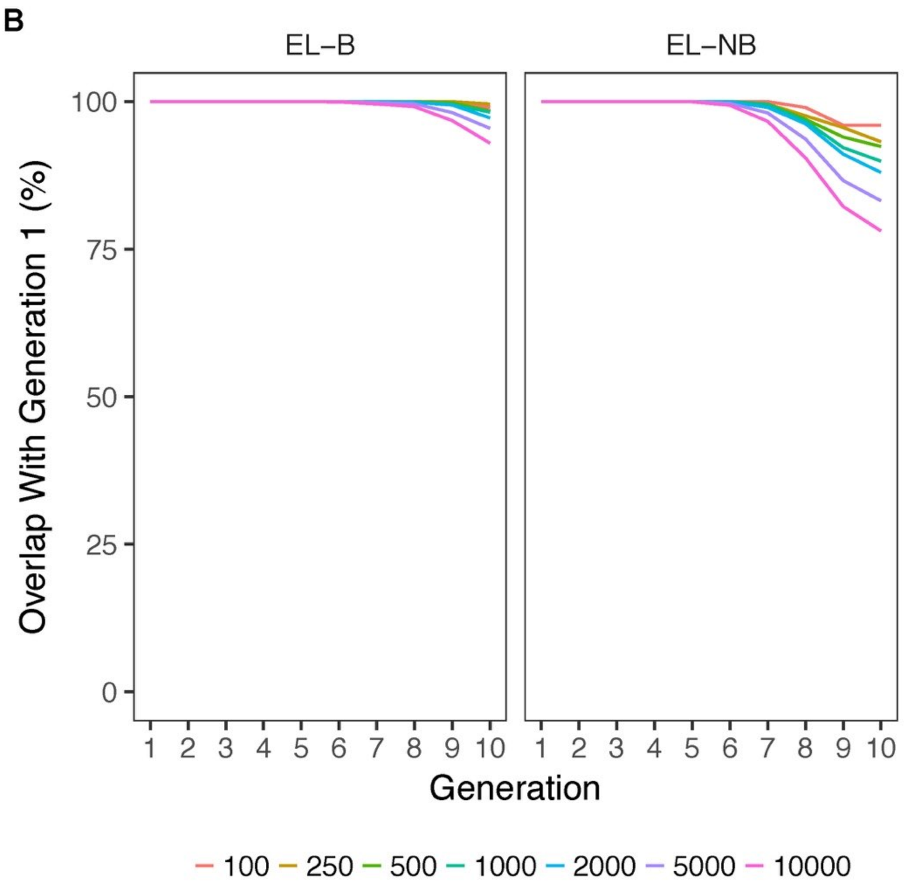

Supplementary Figure 4B. Sequence composition of EL-NB and EL-B after subsequent generations of PCR amplification. The plots show the percent overlap between top sequences at different read count cut-offs (100, 250, 500, 1000, 2000, 5000, 10000) in generation 1 and the following 9 generations. Only the perfect match between sequences was allowed in this analysis. For the 10000-sequence cut-off, 93 percent of sequences were retained for EL-B at generation 10. For EL-NB 78.1% of sequences were retained under these conditions. The amplification of the libraries by generation 4 provides enough material for 10^9 tests.

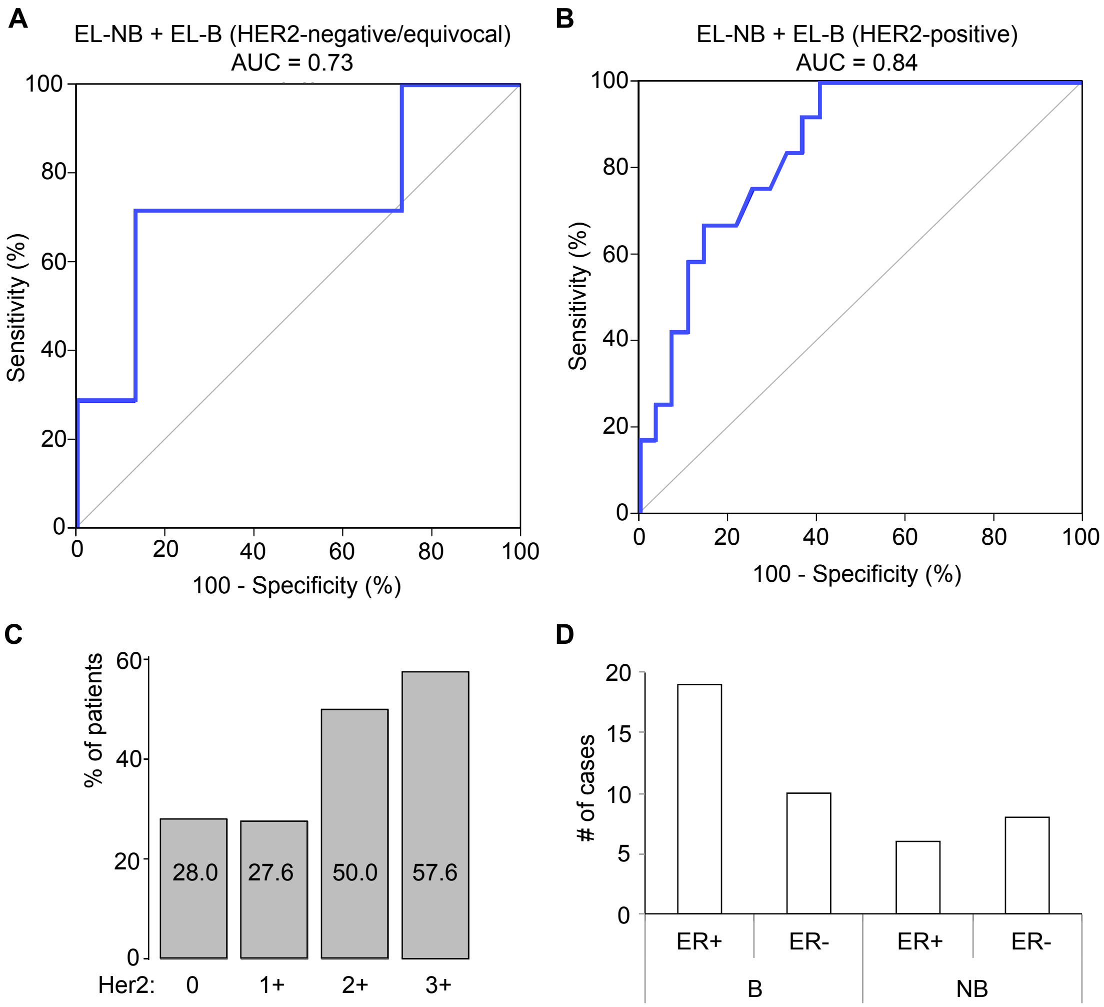

Supplementary Figure 5. Receiver operating characteristic (ROC) curves for differentiation between patients, benefiting and not from C+T or T regimens, using combined histological scores from libraries EL-NB and EL-B PLP staining in the test set, shown separately in the group of 22 HER2-negative/equivocal cases (**A**) and in the group of 39 HER2+ cases (**B**). **C**. Bar graph summarizing the percentages of patients in the PLP positive group according to their IHC-based anti-HER2 antibody staining intensities. **D**. Number of ER-positive (ER+) and ER-negative (ER-) cases within the sets of B and NB cases. The chart shows that in the tested population, benefit from trastuzumab does not correlate with hormonal status.

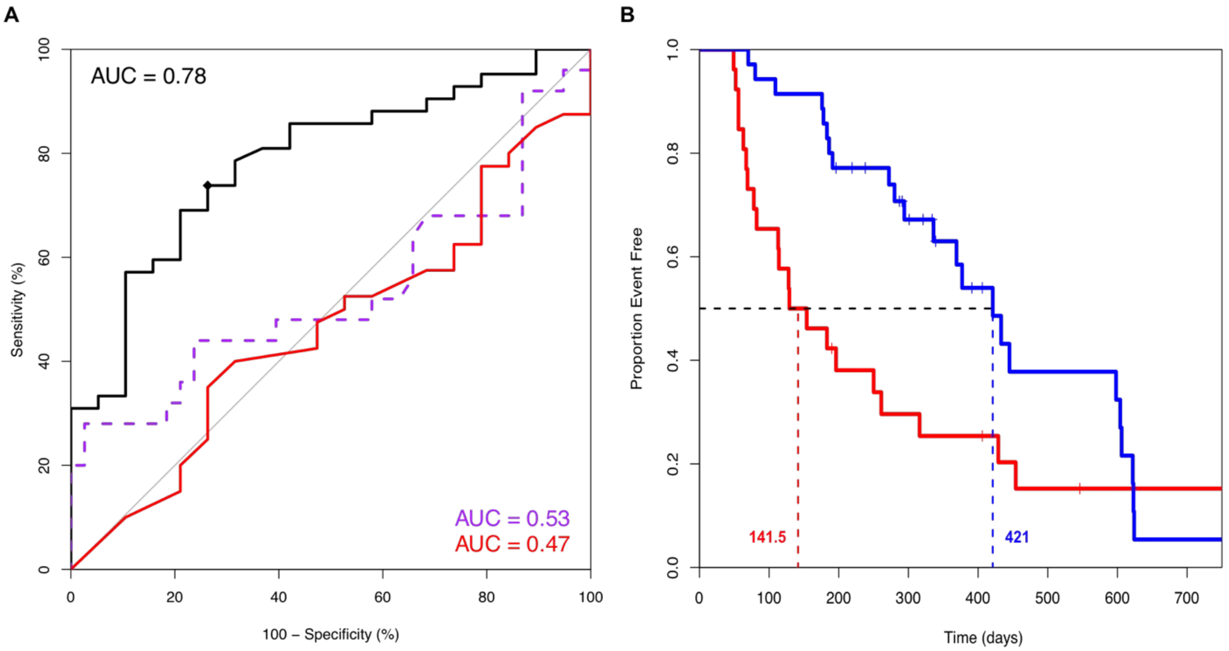

Supplementary Figure 6. Kaplan-Meier curve of C+T- or T-treated breast cancer patients stratified by polyligand profiling after 10-fold cross validation still give significant values in differentiating test-positive from test negative patients. **A**. Same curves shown in Fig. 4C, in which the shortest distance between the ROC curve to point (specificity and sensitivity = 100%) determines the new cutoff of test positive and negative after 10-fold cross validation (black dot; Sensitivity: 78.6%; Specificity: 73.7%). The black curve represents one example of 100 times 10-fold cross-validation that yielded the AUC closest to the mean of all 100 AUCs as the representative cross-validation result. **B**. Kaplan-Meier curve of C+T- or T-treated breast cancer patients stratified by polyligand profiling using 10-fold cross validation. Event is defined as death from cancer or change of trastuzumab-based treatment. Median time of benefit is 421 days for patients tested positive (blue, n = 37, event = 25) and 142 days for patients tested negative (red, n = 24, event = 20). HR = 0.54, 95% CI: 0.21 – 0.70; log-rank p = 0.041. The small vertical lines mark cases that were censored due to absence of treatment follow-up data (**Suppl. Data 4**).
