# Supplementary material for: Poly-ligand profiling differentiates trastuzumab-treated breast cancer patients according to their outcomes
Source: Nat Commun. 2018 Mar 23;9:1219. doi: 10.1038/s41467-018-03631-z (PMC5865185; doi:10.1038/s41467-018-03631-z)
Supplement: Supplementary file 3 — Description of Additional Supplementary Files(PDF 72 kb)(PDF 97 kb) [file 41467_2018_3631_MOESM3_ESM.pdf]

Description of Additional Supplementary Files

File Name: Supplementary Data 1
Description: Cases assignment for enrichment, their treatment history, clinical and demographic details.

File Name: Supplementary Data 2
Description: Comparison between trastuzumab test set and non-qualified set.

File Name: Supplementary Data 3
Description: Next Generation Sequencing data for the unenriched and enriched ssDNA libraries.

File Name: Supplementary Data 4
Description: Test set cases: treatment histories, origin, clinical and demographic details.

File Name: Supplementary Data 5
Description: Staining intensity scores, algorithm score, and the test interpretation for the trastuzumab test set cases.

File Name: Supplementary Data 6
Description: Histological scores for the technical replicates in staining FFPE tissue from 8 patients.
